# *In vitro* comparative study on the friction of stainless steel wires with and without Orthospeed® (JAL 90458) on an inclined plane

**DOI:** 10.4317/jced.52715

**Published:** 2016-04-01

**Authors:** Juan J. Alió-Sanz, Miguel Claros-Stucchi, Alberto Albaladejo, Carmen Iglesias-Conde, Alfonso Alvarado-Lorenzo

**Affiliations:** 1 Professor and Chair, Department of Orthodontics, Complutense University of Madrid, Madrid, Spain; 2 Master’s in Dental Science, Department of Orthodontics, Complutense University of Madrid, Madrid, Spain; 3 Professor in Orthodontics, School of Dentistry, Department of Surgery, University of Salamanca, Madrid, Spain; 4 Professor, Master’s in Orthodontics, University of Alcalá, Madrid, Spain

## Abstract

**Background:**

During the treatment of orthodontics, in the mechanics of slide, there takes place friction, which they reduce the slide of the arch across bracket. Therefore, clinical there takes place an increase of the time of treatment. There are different the technologies that try to reduce this friction, as the self-ligating braces. The purpose of this study was to research the *in vitro* behavior of JAL 90458 as a buffering agent which reduces friction between brackets and stainless steel arch wires of different cross sections and sizes.

**Material and Methods:**

Three types of stainless steel wires with different cross sections and three types of ligatures were used with and without JAL 90458 to measure the friction according to the time and distance traveled by the brackets on an inclined plane with two angulations. The Kruskal-Wallis one-way analysis of variance by ranks was applied to determine the degree of friction between the group using and the group not using the product (P ≤ .05).

**Results:**

Separate analysis of the arch wires, ligatures and angulation with and without the compound revealed statistically significant differences between the groups, showing that friction was reduced significantly when JAL 90458 was used (P ≤ .01). The 0.021x0.025” arch wires and the arch wires attached using elastic ligatures produce the least resistance to sliding among all of those analyzed when the product was not used (P ≤ .05).

**Conclusions:**

The results show that JAL 90458 reduces friction independently of arch wire cross section, type of ligature and angulation of the measuring instrument.

** Key words:**Friction, JAL 90458, arch wires, ligatures, in vitro.

## Introduction

Throughout orthodontic treatment, brackets move along the arch wire or the arch wire moves along tubes and brackets in the alignment and gap-closing stages using a sliding mechanism. During this movement, a negative physical phenomenon occurs which opposes free movement over surfaces and is known as ‘friction’ or ‘resistance to sliding’. This is a deciding factor in treatment length (1-5). Friction is a clinical challenge, particularly with the sliding mechanism, and is the result of physical and biological parameters (6). Friction is also produced by using elastic pieces or metal ligatures that tie the arch to the bracket (7). It can also depend on other factors, such as surface texture (stick-slip phenomenon) (8,9), the friction coefficient, (10) the angulation formed between the axis of the wire and the bracket slot (5,11-13) and the degree of plastic and elastic deformation of the wire (binding and notching), etc (14,15).

Friction increases the forces needed to move a tooth, causing periodontal damage, dislodging of brackets and loss of anchorage (5,16,17). When teeth are moved in the mesial or distal direction, unwanted rotations or angulations of the teeth are produced, which can reduce the applied force by more than 50% due to friction (16). Studies show that friction increases as arch section and strength increase and it is related to the type of ligation used (5,6,18). The arch wire-slot angulation forms a critical contact angle, bringing about a passive or active configuration according to the final degree of angulation produced. The greater the final angulation, the more friction that is produced, tripling every five degrees as the inclination angle increases (7,18,19). There are also second and third order bends (8,13,20). Movement of the teeth while chewing can also be a source of friction at a given moment (14,17). In any event, the friction coefficient is greater for rectangular wires than for round wires and increases with wire thickness (9,21,19). It also increases with the use of esthetic materials (ceramic brackets) (22). Meanwhile, some researchers believe saliva acts as a lubricant and decreases rubbing, while others consider it to actually be an adhesion factor, meaning it therefore hinders sliding (1,21,23).

The development of new self-proclaimed low friction or zero friction materials and techniques that promise to reduce treatment length are based on changing the bracket design and surface structure of the arch wire. This can be done with ion implantation (19,24-26), Poly (Chloro-P-Xylylene) coating, hot nitrogen diffusion (19), alumina crystalline, gas nitriding (27), carbon coating, and plasma (26) and Teflon (16) deposition. However, these changes in the arch wire structure could alter its fundamental properties, as in the case of Nickel-Titanium wire implanted with ions, which changes the elastic recovery of the wire.

A new compound called JAL 90458 (Orthospeed®, Madrid, Spain) is currently being studied. Its colloid consistency as a gel coats surfaces that come into contact with it, neutralizing the forces of friction by acting as a buffer (20). The objective of this study was to measure the resistance to sliding according to the time and distance traveled by a stainless steel bracket on stainless steel arch wires of various sizes and transversal cross sections with and without JAL 90458 on an inclined plane in order to prove that this compound reduces friction in the arch-bracket couple.

## Material and Methods

**Figure 1:**
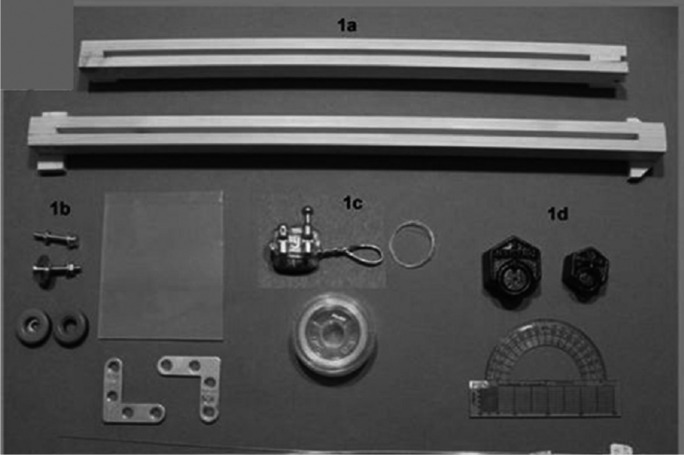
a) Wooden boards of the inclined plane; b) Rubber wheels; c) Bracket with metal ligature; d) 150-gram weights.

**Figure 2:**
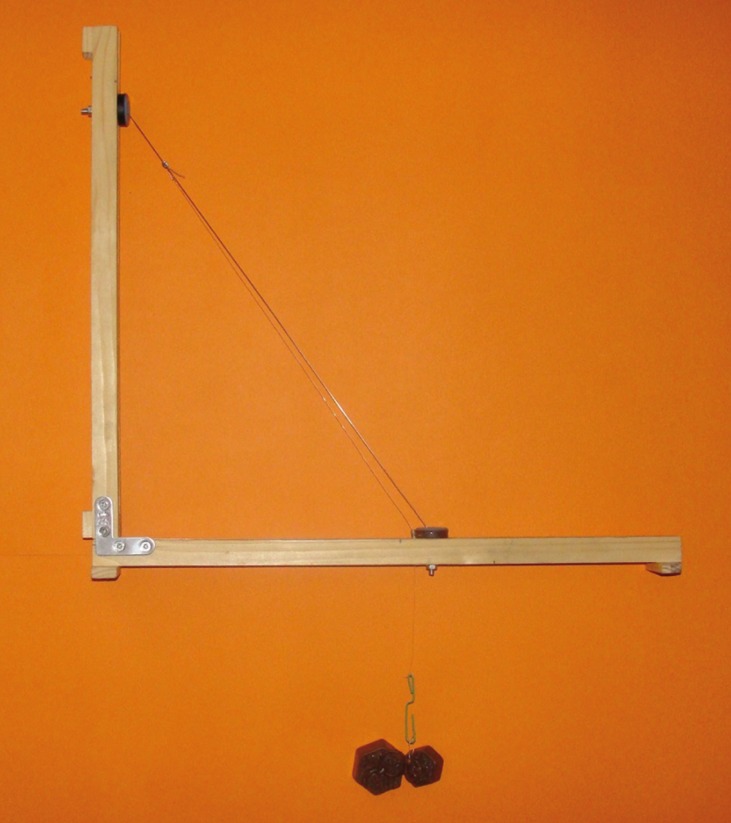
Inclined plane.

For the purposes of this study, we used a 35x40cm inclined plane (Figs. 1,2) with a 0.5 cm groove in the middle where two rubber wheels can roll. One wheel goes in the base and the other in the mast, which will hold the wires at different angulations (45º and 60º) through little loops made of rigid 0.010” stainless steel ligatures attached to each wheel. A small metal tube, measuring 0.8 cm long and 0.08 cm in diameter, was placed on the wheel in the base. A channel measuring 0.04 cm in diameter, which is held by rigid 0.010” ligature, will act as a guide for a 0.25 mm-diameter and 50cm-long nylon line, which will be used to pull the bracket.

Each wire was attached to the loops in the wheels made of 0.010” steel ligature wire. Another 0.010” steel wire loop was attached to each bracket with photopolymerized resin (Tetric® Ceram, Ivoclar Vivadent S.A. México). The 50-cm nylon line was tied to this loop in the bracket, then, from there, it was threaded through the small metal tube on the aforementioned wheel at the base and finally tied to some 150-gram weights. A protractor was adapted to act as a guide to measure the different angulations of the wires.

Six stainless steel 304 VAR arch wires (Densply Gac-Orthodontic, Lima, Peru) were used with the following characteristics: two 0.020” round arch wires, two 0.019x0.025” rectangular arch wires and two 0.021x0.025” rectangular arch wires. Each wire was 38cm long. Synergy® (Rocky Mountain Orthodontics, RMO, Denver, EE.UU.) straight wire low friction 0.022” slot size AISI 316 stainless steel brackets were used on the right-hand maxillary central incisors.

We used the following ligatures: 0.010”, 5-cm-long, AISI 304VAR stainless steel ligature (Densply Gac-Orthodontic), conventional 0.120” elastomeric ligatures (Ormco Co, California, EE.UU), and 0.120” silicone injected low friction ligatures (RMO). Two types of JAL 90458 were used: Compound 1, with a more liquid consistency, and Compound 2, more viscous in nature (20).

-Methodology

1.- The wires were placed at 45º and 60º degrees respectively in the measuring instrument starting with the lowest caliber wires (0.020”) and proceeding with the higher caliber wires (0.019x0.025” and 0.021x0.025”). The time and distance traveled by the bracket was recorded for each of the steel wires of differing calibers in combination with the three types of ligatures and angula-tions. This was done without using JAL 90458.

2.- JAL 90458 was applied immediately afterwards on the surface of the wires and brackets (compound 2) and on the ligatures (compound 1) and measurements of time and distance were taken.

We used 45º and 60º angulations due to the influence of the critical contact angle in second-order angulations. To ensure accurate measurements, a standard weight of 150 grams was used, since a lesser weight would not produce any movement and a greater weight would make measuring the distance more difficult due to the speed at which the bracket would slide. One bracket for each wire caliber was used with and without the compound. The metal ligatures attached the wire with a total of 5 half turns for each test (the procedure was always carried out by the same person to avoid the bias of different ligation strengths). The time and distance measurements were single blind in a dry environment at room temperature using a digital calibrator.

-Determining Sliding Resistance

The maximum time for the bracket to slide the maximum distance from point A to point B along each wire was measured. The maximum time allotted was 30 seconds and the total distance was 30cm. If the bracket did not complete the 30-cm distance, a sliding time of 30 seconds was given.

-Statistical Analysis

The data were analyzed using the SAS 9.1 statistical program. The descriptive statistics of numeric independent variables for this test included the average and median of the time and distance of all the arch-bracket couples. The Kruskal-Wallis one-way analysis of variance by ranks for independent samples was applied to compare the different cross sections of wire, the different types of ligature and the different angulation. Statistical significance was (*P* ≤ .05).

## Results.

The tables we have included show the results obtained with and without JAL 90458 with the three different types of wire used in the study (0.20”, 019.025” and 021.025”).  Table 1 shows the results with and without the product, comparing the three types of wire used with the three different types of ligatures (conventional elastic, low friction elastic and metal), all in relation to the time the bracket took to slide along the wire. We observed that without JAL, the longest period of time was registered with the 021.025” wire and a conventional elastic ligature. With JAL 90458 the time used was much lower. The comparison between the two groups (with and without JAL 90458) was highly significant.

**Table 1:**
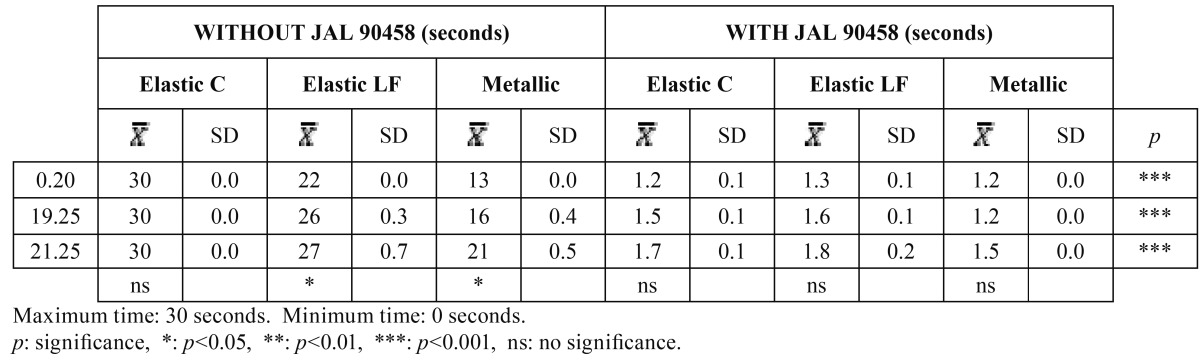
Results of time slide to 60º, with and without JAL 90458.

 Table 2 shows the same results but with a 45º angulation. As expected, the times were shorter at a greater angulation for the results without JAL 90458. However, when we applied the product, the results were not significantly different from those obtained at a 60º angulation. Furthermore, there were no differences with respect to the conventional elastic ligature, which continues to be the one with the greatest degree of sliding resistance. The differences between the two groups were also highly significant.

**Table 2:**
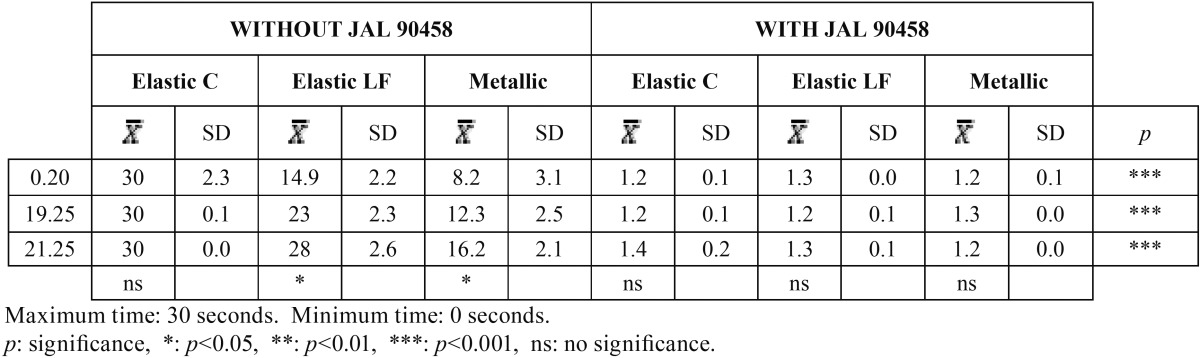
Results of time slide to 45º angulation, with and without JAL 90458.

 Table 3 shows the results obtained with and without JAL 90458 on conventional elastic ligatures in relation to the time the bracket took to move a certain distance. Without JAL 90458 the bracket did not slide at all. With the product, we observed that as the wire section increased, the distance traveled decreased. Highly significant differences were found between the two groups.

**Table 3:**
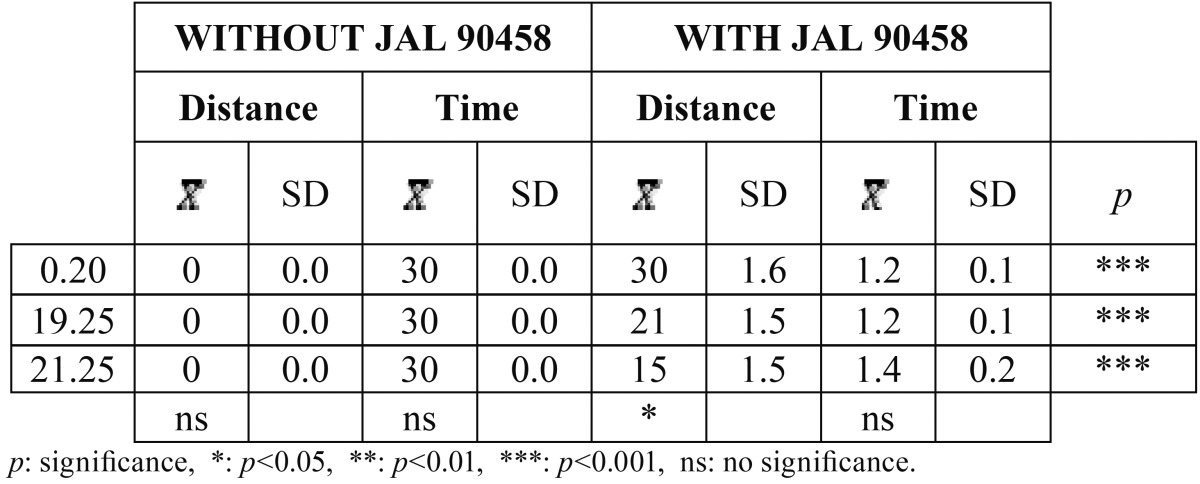
Distance covered in the different types of wires with conventional elastic ligatures, in relation to time and with and without JAL 90458, to 45º of angulation.

When low friction elastic ligatures were used ( Table 4), the bracket did move, even if JAL 90458 was not applied. In all cases, the differences between the two groups were also statistically significant.

**Table 4:**
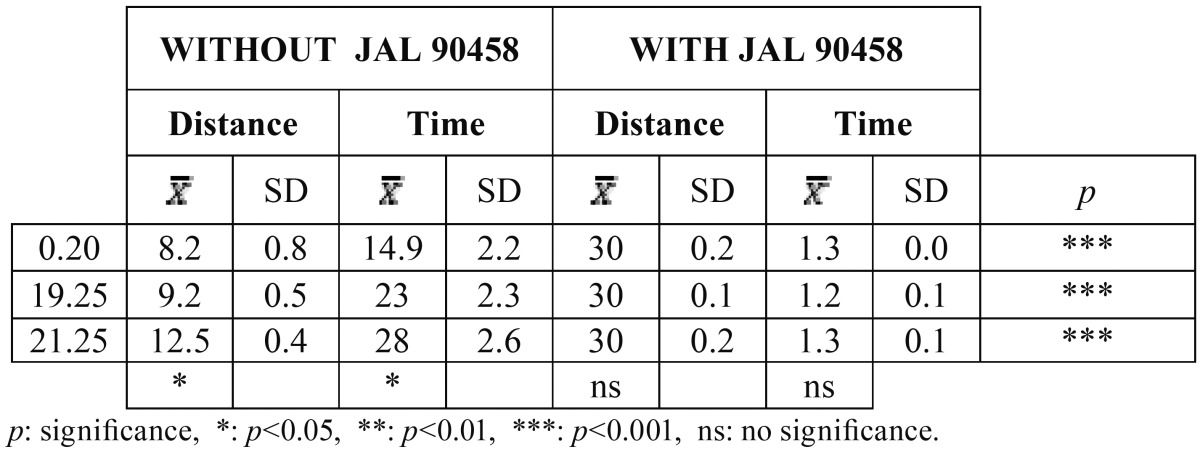
Distance covered in the different types of wires with low friction elastic ligatures, in relation to time and with and without JAL 90458, to 45º of angulation.

## Discussion

In order to find solutions to sliding resistance, researchers are carrying out many different studies in the areas of biomechanics and mechanotherapy, thus favoring the development of new materials. However, the experiments carried out with techniques intended to reduce sliding resistance continue to encounter problems (19,25,26). With JAL 90458 we find friction reduction to be very high, showing substantial statistically significant differences. This efficiency in reducing friction was shown for every type of ligature used. Some studies have not found differences regarding the types of ligature. Redlich (27), for example, did not find any differences with respect to the type of ligature when comparing conventional brackets to self-ligating brackets. In this case, we witnessed the lowest levels of friction when using metal ligatures, followed by low friction elastic ligatures. This difference could be due to the method used to ligate the metal ligatures. Our results concur with those obtained by Fortini *et al.* (28).

Another important quality of this product is that, in addition to reducing friction, it does not change the properties of the wires (20), only acting as a buffering agent between the wire and the bracket, preventing rubbing between the two by sealing the rough surfaces. In contrast, ion implanted wires (25) modify the springback quality of Niti and affect the release of force of TMA® wires.

Most studies done on wire cross sections have found that the bigger the wire, the greater the friction (5,7,11,12,14,16,17,24,29-31). Our study coincides with these results. This difference was most evident when JAL 90458 was not applied. However, when we used the product, the differences between wire sizes were not so pronounced.

## Conclusions

JAL 90458 significantly reduced sliding resistance on all the stainless steel wire-bracket couples. JAL 90458 also significantly reduced friction on all the ligatures studied.

The transversal cross section and caliber of the wires did not have a great influence on friction increase when JAL 90458 was used compared to the results obtained in the group that did not use the product.

When JAL 90458 was not used, the metal ligature created the least amount of friction.

The wire-bracket couple sliding time when JAL 90458 was used was always lower than when the compound was not used. The distance traveled by the brackets was always greater when JAL 90458 was used than when the test was carried out without applying the compound.
